# Binding Performance of Human Intravenous Immunoglobulin and 20(*S*)-7-Ethylcamptothecin

**DOI:** 10.3390/molecules23092389

**Published:** 2018-09-18

**Authors:** Yong-Chun Liu, Ying-Ying Li, Xiao-Jun Yao, Hui-Li Qi, Xiao-Xia Wei, Jian-Ning Liu

**Affiliations:** 1 College of Chemistry and Chemical Engineering, Longdong University, Qingyang 745000, China; liyingyinghx@163.com (Y.-Y.L.); qihuili33@sohu.com (H.-L.Q.); xiaoxia9533@163.com (X.-X.W.); ldxyljn@163.com (J.-N.L.); 2 Longdong University & FLUOBON Collaborative Innovation Center, Longdong University, Qingyang 745000, China; 3 College of Chemistry and Chemical Engineering, Lanzhou University, Lanzhou 730000, China; yaoxj@lzu.edu.cn

**Keywords:** intravenous immunoglobulin (IVIG), camptothecin analogues (CPTs), 7-ethyl-camptothecin, drug delivery, protein-drug interaction

## Abstract

A previous study showed that intravenous immunoglobulin (IVIG) could preserve higher levels of biologically active lactone moieties of topotecan, 7-ethyl-10-hydroxycamptothecin (SN-38) and 10-hydroxycamptothecin at physiological pH 7.40. As one of camptothecin analogues (CPTs), the interaction of 7-ethylcamptothecin and IVIG was studied in vitro in this study. It was shown that the main binding mode of IVIG to 7-ethylcamptothecin was hydrophobic interaction and hydrogen bonding, which is a non-specific and spontaneous interaction. The hydrophobic antigen-binding cavity of IgG would enwrap the drug into a host-guest inclusion complex and prevent hydrolysis of the encapsulated drug, while the drug is adjacent to the chromophores of IgG and may exchange energy with chromophores and quench the fluorescence of the protein. Also, the typical β-sheet structure of IVIG unfolded partially after binding to 7-ethylcamptothecin. Additionally, the binding properties of IVIG and six CPTs with different substituents at *A*-ring and/or *B*-ring including camptothecin, topotecan, irinotecan, 10-hydroxycamptothecin, 7-ethylcamptothecin and SN-38 were collected together and compared each other. Synergizing with anti-cancer drugs, IVIG could be used as a transporter protein for 7-ethylcamptothecin and other CPTs, allowing clinicians to devise new treatment protocols for patients.

## 1. Introduction

**Scheme 1:**
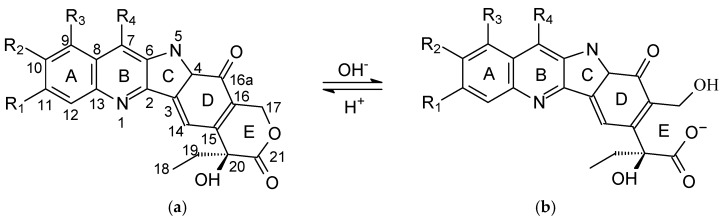
The reversible hydrolysis equilibrium of the lactone (**a**) and the ring-opened carboxylate form (**b**) of CPTs. Camptothecin (CPT): R_1_ = H, R_2_ = H, R_3_ = H, R_4_ = H; Topotecan: R_1_ = H, R_2_ = OH, R_3_ = CH_2_N(CH_3_)_2_, R_4_ = H; Irinotecan: R_1_ = H, R_2_ = 
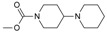
, R_3_ = H, R_4_ = CH_2_CH_3_; 10-Hydroxycamptothecin: R_1_ = H, R_2_ = OH, R_3_ = H, R_4_ = H; 7-Ethylcamptothecin: R_1_ = H, R_2_ = H, R_3_ = H, R_4_ = CH_2_CH_3_; SN-38: R_1_ = H, R_2_ = OH, R_3_ = H, R_4_ = CH_2_CH_3_.

Camptothecin analogues (CPTs) undergo a pH-dependent reversible hydrolysis in aqueous solution due to their chemically unstable *E*-rings. Under acidic conditions (pH ≤ 4) as shown in Scheme 1, the lactone moeity with (20)*S*-configuration is predominant in CPTs, which can interact with DNA-enzyme complex, and present essential biological activities and effective drug activity against several malignant tumors, while at pH ≥ 10, the carboxylate form presents exclusively, which has little topo I inhibitory activity and even presents toxicity [1,2,3]. Temperature changes change the rate of interconversion between moieties, but do not affect the reversible hydrolysis equilibrium [4]. However, CPTs present lower levels of lactone in equilibrium at pH 7.4 either in phosphate buffer solution (PBS) alone, HSA in PBS, plasma, and human whole blood system [5,6,7].

Additionally, multidrug resistance of cells to CPT drugs is a serious problem in clinical use, however, the mechanisms underlying and acquired cellular resistance to CPTs are not yet clear at present [8]. The adenosine triphosphate–binding cassette protein ABCG2 (breast cancer resistance protein [BCRP], mitoxantrone resistance [MXR]) causes multidrug resistance in cancer cells and may have an important function in physiological protection of various tissues against toxic agents. ABCG2 was first cloned from the placenta, where it is most abundantly expressed [9]. The overexpression of ABCG2 is observed in certain drug-resistant cell lines and tumors, providing a special multidrug-resistant phenotype in these cancer cells [10], which indicates its possible importance in the multidrug-resistant phenotype of various cancer cells [11]. The ABCG2 protein has only one nucleotide binding (ABC) and one transmembrane domain and most probably works as a homodimer in the plasma membrane [12]. The substrate specificity of ABCG2 partially overlaps with the other major multidrug resistance ABC transporters, MDR1 and human multidrug resistance protein 1 (MRP1); the compounds transported by ABCG2 are also large, hydrophobic molecules, including mitoxantrone, topotecan, flavopiridol, methotrexate, and Hoechst 33342 [13,14,15]. Using transduced clonal cell lines expressing varying levels of ABCG2 finds that ABCG2 expression confers resistance to mitoxantrone and topotecan, but not to idarubicin [16]. Studies reveal that yeast SCT1 mutants result in camptothecin resistance and the ATP-binding cassette transmembrane transport proteins involve in cellular multidrug resistance to CPT [17], which is consistent with a model of decreased intracellular concentrations of camptothecin resulting from the increased expression of the SNQ2 transporter. However, a monoclonal antibody, named 5D3, prepared by immunizing mice with intact mouse fibroblasts expressing the human ABCG2 and made commercially available (eBioscience) [18], can specifically react with the human ABCG2 protein on the cell surface [15] to inhibit the Hoechst 33342 dye transport function of ABCG2 in intact cells [16]. It is also found that the interaction of 5D3 with ABCG2 is strongly dependent on the modulation of the multidrug transporter protein, thus, 5D3 binding to an extracellular ABCG2 epitope is conformation-sensitive. Similar antibodies have already been prepared against the human MDR1 multidrug transporter [19,20]. In the case of MDR1, several of the mAbs reacting with extracellular epitopes are found to inhibit the transport function of the protein, and the reactivity of one of these antibodies, UIC2, is reported to depend on the conformation of the MDR1 protein [19,21,22]. In fact, genes of ABCG2 have over 40 single nucleotide polymorphisms [23,24], it’s hard to identify these nucleotide polymorphisms completely by a single monoclonal antibody. However, human IVIG (pH 4, IgG ≥ 95%) contains 10^8^ specific molecules of IgG, and traces of IgA and IgM [25], may have superiorities in identifying more genetic variants of ABCG2, inhibiting the transport and function of ABCG2 proteins as 5D3. At present, a high quality of 5–10% IVIG solution (pH 4) has been achieved with a high recovery rate from human plasma and used in clinic [26,27,28], this acidic condition itself is just suitable to maintain the lactone structure of CPTs initially. 

On the other hand, IVIG has been used in the treatment of primary and secondary antibody deficiencies for over 35 years [29]. Recently, great attention has been paid to the potential use of IVIG as an adjuvant anti-neoplastic agent [30,31,32]. Studies on the anticancer alkaloid vindesine (VDS) conjugates of the anti-carcinoembryonic antigen (CEA) antibodies against a colorectal tumor xenograft reveal that VDS−11.285.14 (IgG1) conjugate can produce an almost complete and lasting suppression of tumor growth, and it is considerably less toxic than free VDS in Balb/c mice [33]. There are different transfer mechanisms between the drug-protein conjugates and free drugs transferred into cells, and the conjugates can reduce the toxicity of drugs, resulting in a prolongation of survival time [34]. IVIG has been widely used in diseases involving uncontrolled C activation, and it suppresses C activation by decreasing alternative pathway amplification via C3b-containing complexes [35]. Study on the efficacy of an IgG F(ab)_2_ preparation against taxol-induced C activation reveals that at a therapeutically relevant dose level (10 mg/mL), this Fc-depleted IgG causes significant suppression of taxol-induced SC5b−9 formation, but replacing IVIG with 10 mg/mL HSA causes no inhibition of taxol-induced rise of SC5b−9 [36]. Also, IVIG may stimulate the production of IL−12, an anti-tumor and anti-angiogenic cytokine, enhance NK cell activity [31], and can decrease the level of matrix metalloproteinase−9 (MMP−9) expression in the U937 monocyte line with a decrease in the m-RNA level of MMP−9, a vital step in the invasion of metastatic cancer cells. Moreover, IVIG presents biologically natural and intact function [25], synergizing with anti-cancer drugs, so IVIG may serve as an adjuvant therapy in cancer due to its anti-neoplastic properties that may synergize with more specific MMP inhibitors and other anti-cancer drugs [37,38]. Factually, as of 1 May 2017, 74 antibody-based molecules have been approved by a regulatory authority in a major market. Additionally, there are 70 and 575 antibody-based molecules in phase III and phase I/II clinical trials, respectively. These total 719 antibody-based clinical stage molecules include 493 naked IgGs, 87 antibody-drug conjugates (ADCs), 61 bispecific antibodies, 37 total Fc fusion proteins, 17 radioimmunoglobulins, 13 antibody fragments, and 11 immunocytokines [39]. New uses for these antibodies are being discovered each year. ADCs target a cytotoxic drug to a tumor to kill cancer cells while lowering the systemic exposure of the active moiety, with the goal of increasing the size of the efficacy/toxicity window of highly toxic anti-tumor drugs [40,41,42]. In 87 clinical stage ADCs, there currently are three approved ADCs, nine in phase III development, and another 75 in phase I/II clinical development. The three approved ADCs include Mylotarg^®^ (2000, withdrawn in 2010), the CD30-targeting Adcetris^®^, and the ERBB2 (Her2)-targeting Kadcyla^®^. These 87 clinical stage ADC molecules are directed against at least 53 different known targets, although a few have not been disclosed. The most targeted cell surface receptors currently are ERBB2 and CD19 (4 ADCs against each), and CD33, CD22, and MSLN (mesothelin) (three ADCs against each). There are 16 known different classes of drugs incorporated into clinical stage ADCs, 11 of which are small molecule classes and five of which are protein-based. The most widely used drug class incorporated into clinical stage ADCs are the auristatins (employed 31 times), followed by the maytansanoids (in 16 ADCs), and benzodiazepines (used in nine ADCs). Of the biologics, *Pseudomonas exotoxin* PE38 is incorporated into four ADCs. Moreover, over the past decade there has been a significant shift from discovery and development of basic antibodies, e.g., naked IgG1 isotype antibodies with no additional engineering other than perhaps humanization and affinity maturation, to more sophisticated forms of antibodies in all kinds of shapes and sizes. 

Previously, the interaction of IVIG and several (20)*S*-configuration CPTs have been studied in vitro [43,44,45] and study reveals that IVIG can maintain higher circulatory levels of lactone moieties of three CPTs at physiological pH 7.40 compared to HSA [45]. In this study, as one of camptothecin analogues, the binding performance of 7-ethylcamptothecin and IVIG will be investigated in vitro, further the binding properties of IVIG and six (20)*S*-configuration of CPTs with different substituents at *A*-ring and/or *B*-ring will be collected together and compared each other. Using synergization of anti-cancer CPTs with IVIG, clinicians may develop new treatment protocols for patients.

## 2. Results and Discussion

### 2.1. Fluorescence Quenching Study

**Figure 1:**
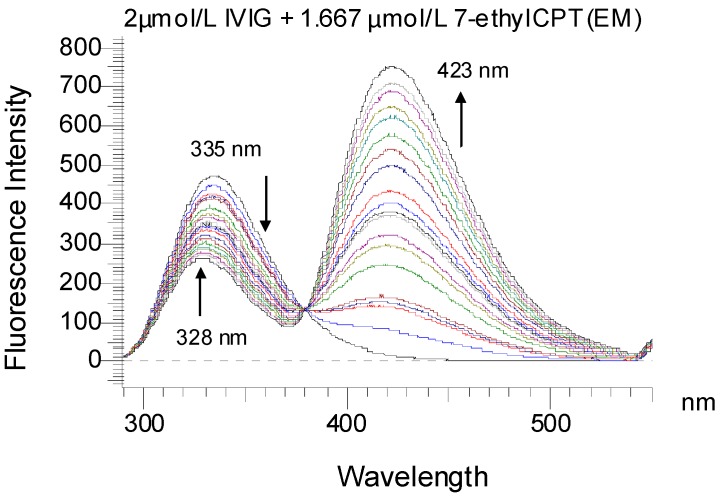
Fluorescence spectra of IVIG of 2.0 μM free (the top spectrum at 335 nm) titrated by successive addition of 7-ethylcamptothecin solution (1.667 μM) to give a final concentration of 33.33 μM (the bottom spectrum at 328 nm) with the interval of successive addition by 5 min; λ_ex_ = 280 nm, λ_em_ = 335 nm; pH 4.0 and 310 K.

Figure 1 shows the fluorescence spectra of free IVIG (the top spectrum at 335 nm) and after successive addition of 7-ethylcamptothecin solution with a characteristic emission maximum at 335 nm when exited at 280 nm. However, 7-ethylcamptothecin itself presents a strong fluorescence emission band at 423 nm. The study on protein−protein and protein−peptide interactions by fluorescence spectroscopy reveals that the emission wavelength of the Trp fluorescence blue-shifted from 353 to 325–333 nm indicates a movement of Trp from a solvent water exposed to a hydrophobic environment, and the quenching effect is markedly distance dependent, as it only influences the Trp residue of the bound peptide (≤7 Å) but has little effect on the two Tyr residues (≥10 Å) [46]. This blue shifting of the IVIG emission band from 335 nm toward to 328 nm indicates the chromophores of protein are involved in a more hydrophobic environment when binding to 7-ethylcamptothecin which indicates that more and smaller water molecules located in hydrophobic cavity of IVIG may be replaced by 7-ethylcamptothecin and expelled by hydrophobic interaction. In fact, this is a general phenomenon for other CPTs but not palpably for 10-hydroxycomptothecin and SN-38, however, it is desirable that CPTs avoid hydrolysis in aqueous solution. The fluorescence spectra of IVIG in the presence of 7-ethylcamptothecin are similar to each other at pH 4.0, 7.4 and 10.0 (plots are not shown). Additionally, all of the analogues after added to IVIG can cause a decrease in emission at 335 nm, and increases at 422~442 nm for camptothecin, irinotecan, 7-ethylcamptothecin, and an increase at 525 nm for topotecan, but almost no fluorescent emission for SN-38 and 10-hydroxycamptothecin, which is due to the inner fluorophore structures and the effects of hydroxyl substituent at-10, respectively. However, it indicates that the interaction has taken place between IVIG and every analogue.

**Figure 2:**
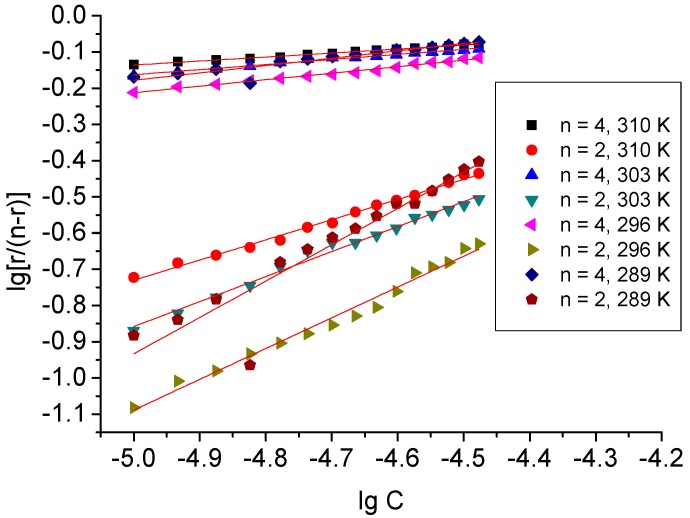
Sips plots of IVIG binding to 7-ethylcamptothecin at pH 4.0 and different temperatures.

As one of classical methods illustrating the interactions between biological macromolecules and small drugs, the Sips procedure was selected for evaluating the interaction parameters of IVIG with 7-ethylcamptothecin and other analogues as shown in Figure 2. The Stern-Volmer dynamic quenching constants *K*_SV_ (10^4^ M^−1^) and the quenching rate constants *K*_q_ (10^12^ M^−1^·s^−1^) are calculated and collected in Table 1, in which τ_o_ (the average fluorescence lifetime for biological macromolecule) is taken as 10^−8^ (s^−1^) [47,48]. Other typical interaction parameters of IVIG with 7-ethylcamptothecin and other analogues are also summarized in Table 1. The obtained *K*_q_ (10^12^ M^−1^·s^−1^) is larger than the maximum value of dynamic quenching rate constant of bimolecular diffusion collision, *K*_q(max)_ (2.0 × 10^10^ M^−1^·s^−1^), indicating that there exists a static quenching mechanism between the fluorophores and each CPT analogue arisen from the formation of a dark complex between the fluorophores and quenching agent. So, the dynamic quenching constant *K*_SV_ can be interpreted as the association or binding constant of the reaction of complexation in this case [43]. On the other hand, most of the binding sites of IgG for antigen/semiantigen are located in the complement-determining region (CDR) of F(ab)_2_ (*n* = 2), however, the crystallizable fragment (complement-bing site, Fc) regions also considered to a certain extent to have similarity to F(ab) and can bind to smaller molecules [49,50,51], though there are more potential binding sites for IgG binding to antigen/semiantigen [52,53,54]. It is found that there are slightly better Sips linear correlations, higher binding constants and higher affinity heterogeneity index of protein (α) when *n* = 2 than *n* = 4, which indicates there are two stronger but four mainly binding sites of IVIG binding to these CPTs and all of the interactions of IVIG with these CPTs are of non-specific with *K*_o_ (binding constant or average affinity constant) at 10^3^~10^4^ M^−1^, and weak interactions in comparison to the stronger ligand-protein binding with the affinity constants at 10^6^~10^8^ M^−1^ [55]. However, studies on the interaction of IgG with chlorogenic acid, ciprofloxacin, forsythoside, resorcinol phthalein and pefloxacin by fluorescence spectroscopy reveal that all the modified Scathard linear correlations are better when *n* = 4 than those when *n* = 2, indicating that the mainly binding sites of IgG for these five drugs are four, where sites of F(ab)_2_ and Fc have almost the same binding abilities for these drugs [49]. Additionally, camptothecin presents a stronger binding than the others, may be due to the hydrophobicity and size of the molecule that is more adaptive to the shallow hydrophobic antigen/semiantigen-binding cavity of IVIG.

### 2.2. Binding Mode Study

Typical thermodynamic parameters of IVIG interacting with CPTs are calculated and collected in Table 2. According to the relationship of binding modes with the values of typical thermodynamic parameters, enthalpy Δ*H*^o^ and entropy Δ*S*^o^ [56], the main interaction types of IVIG and camptothecin, topotecan, irinotecan, 10-hydroxycamptothecin, 7-ethylcamptothecin, SN-38 are hydrophobic interaction, Van der Waals force and hydrogen bonding, electrostatic interaction, electrostatic interaction, hydrophobic interaction, Van der Waals force and hydrogen bonding, respectively, owing to their different substituents at *A*-ring or/and *B*-ring. It is found that entropy driving plays a main role for camptothecin and 7-ethylcamptothecin, while enthalpy plays the main role for other analogues, however, the binding processes of IVIG to these CPTs are spontaneous as confirmed by the negative values of Δ*G*^o^. As for irinotecan and 10-hydroxycamptothecin, the main binding modes of both are electrostatic interactions, possibly due to their partly ionized structures in solution at pH 4.0, however, it can’t be excluded for other analogues in this lower pH environment. Moreover, SN-38 presents the mainly binding modes of Van der Waals force and hydrogen bonding as topotecan, possibly due to the microenvironments of antigen/semiantigen-binding cavity of IVIG. In addition, both the main binding modes of IVIG to camptothecin and 7-ethylcamptothecin are hydrophobic interactions resulting from their hydrophobic properties. It may be suggested that the more hydrophobic the molecule is, the stronger the affinity with which IVIG binds to it, which suggests options for clinicians in terms of medication cycles and dosages. Camptothecin, both hydrochlorides of topotecan and irinotecan, and 10-hydroxycamptothecin have been used in clinical treatment for cancers.

### 2.3. FT-IR Spectroscopy Study

**Figure 3:**
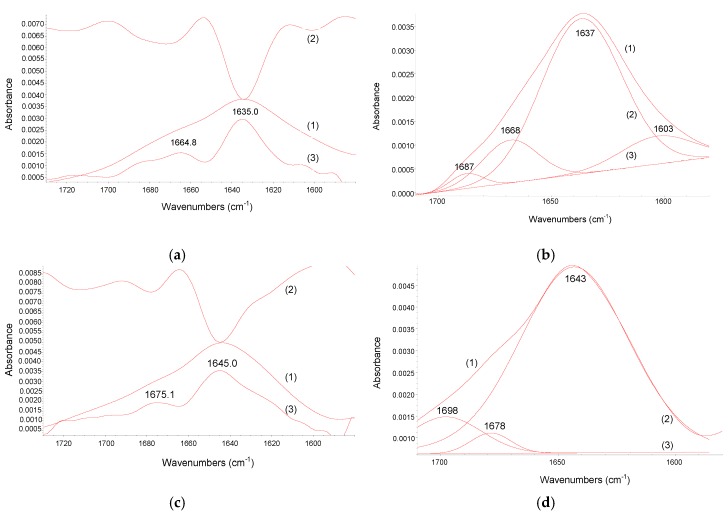

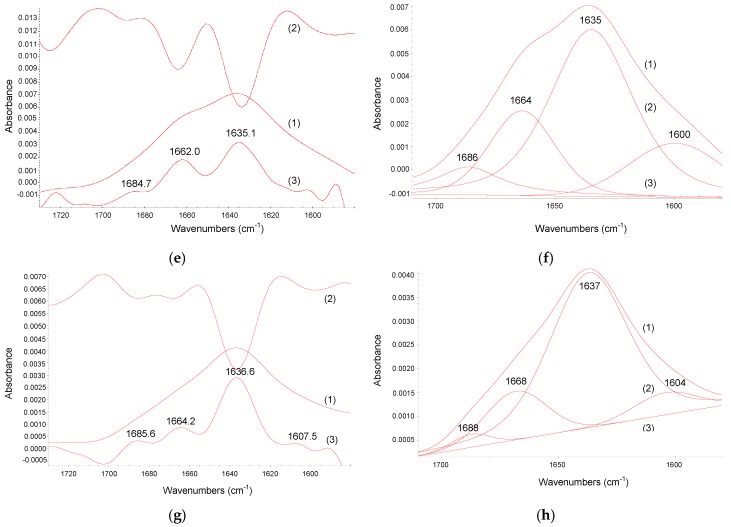
IVIG FT-IR spectra and the curve-fitting procedures of amide I band in the presence of 7-ethylcamptothecin at 296 K. The concentration of IVIG is 2.0 µM. (**a**,**b**) The concentration of 7-ethyl-camptothecin is 4.0 µM at pH 4.0; (**a**) (1), (2) and (3) represent the difference spectra, second derivative resolution enhancement and self-deconvolution, respectively; (**b**) (1), (2) and (3) represent the difference spectra, curve-fitting results and curve-fitting baseline, respectively. The means represented by numbers (1, 2, 3) in parentheses in figures of (**c**,**e**,**g**) are the same as those in (**a**), and the means represented by numbers (1, 2, 3) in the figures of (**d**,**f**,**h**) are the same as those in (**b**). (**c**,**d**) The concentration of 7-ethylcamptothecin is 8.0 µM at pH 4.0; (**e**,**f**): The concentration of 7-ethylcamptothecin is 4.0 µM at pH 7.4; (**g**,**h**) The concentration of 7-ethylcamptothecin is 8.0 µM at pH 7.4.

Since it is more sensitive to the secondary structure [57,58], the FT-IR spectrum of the amide I band of the protein is selected to study the changes of the IVIG secondary structure in the presence of 7-ethylcamptothecin, as shown in Figure 3, then the secondary structural contents of free IVIG and the ones in the presence of 7-ethylcamptothecin and other analogues are estimated and collected in Table 3. Apparently, the typical β-sheet composition of IVIG decreases, i.e., the typical β-sheet structure of IVIG unfolds partially after binding to CPTs, while the remainder, mainly random coil and α-helix, increases in the presence of CPTs whether at pH 4.0 or at pH 7.4. As for the compositions of β-antiparallel and turn, there are irregular changes. However, the remainders present higher compositions in the presence of CPTs, especially higher compositions of random coil, suggesting that the binding of IVIG to CPTs may affects its biological functions, which should be further studied in vivo.

### 2.4. UV-Vis Spectroscopy Study

Based on the distinguishable UV-vis spectra of SN-38, 10-hydroxycamptothecin and topotecan between testing at pH 4.0, 7.4 and 10.0 in PBS (data shown in Table 4), the calculated ratios of lactone to total drug decrease from 90.40%, 86.68% and 34.47% in the absence of IVIG, to 86.47~87.35%, 56.56~68.58% and 29.76~32.71% in the presence of IVIG within the subsequent incubated 60 min at pH 7.40, respectively [43]. However, compared to the interaction of HSA with some CPTs at pH 7.4 either in PBS alone, HSA in PBS, plasma and human whole blood system, IVIG can maintain higher circulatory levels of lactone moieties of these CPTs [5,6,7], which indicates that IVIG preferentially binds the lactone form of the three CPTs and thereby preserves higher circulatory levels of the biologically active species at physiological pH 7.40. Although there are not distinguishable differences in the UV-vis spectra for camptothecin, irinotecan and 7-ethylcamptothecin between testing at pH 4.0, 7.4 and 10.0, it is probably suggested IVIG may also maintain high levels of lactone moieties of other CPTs, which should be further studied by other methods.

### 2.5. Molecular Docking Study

**Figure 4:**
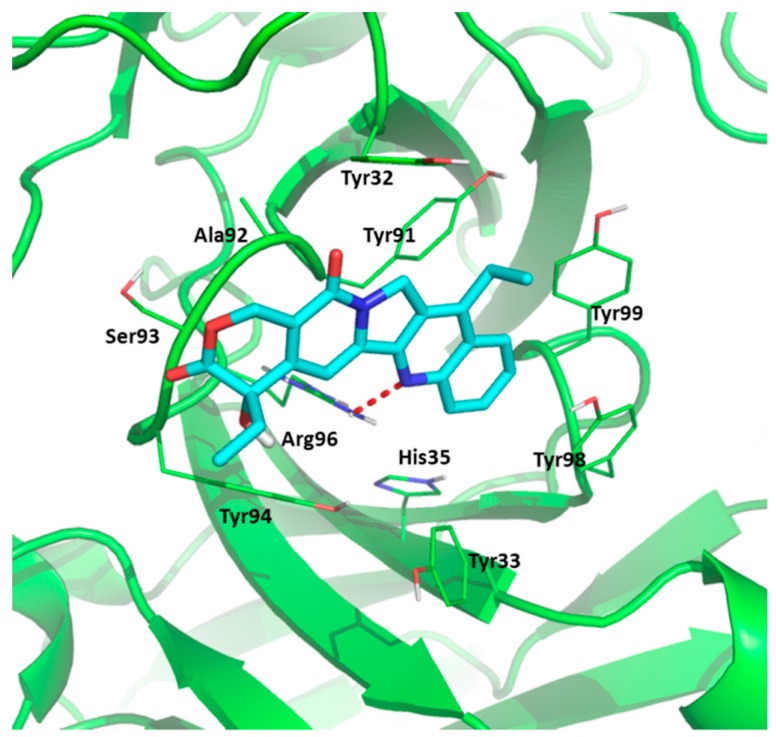
Molecular docking plots of human IgG with 7-ethylcamptothecin. Only residues around 6 Å of the drug are displayed. Hydrogen bonding formation is indicated by dash lines.

Structural mode of human IgG1 reveals that heavy chain variable domain (V_H_ domain) is composed of amino acid residues encoded as 31–37, 51–68, 84–91, 101–110 and 121, while the light chain variable domain (V_L_ domain) is composed of residues encoded as 26–32, 48–55, 90–95 and 107, which may constitute a shallow cavity/cleft (possibly CDR of Fab) as essential binding sites for antigens/semi-antigens [44,45,46,47,48,49]. As shown in the molecular docking plots (Figure 4), the 7-ethyl-camptothecin molecule locates in the antigen-binding cleft composed of V_L_ 26–32, V_H_ 31–37, V_L_ 90–95 and V_H_ 96–99. As for the other analogues, the drug molecules mainly locate in the cleft composed of V_L_ 26–32, V_H_ 31–37, V_L_ 48–55, V_H_ 51–68, V_H_ 84–91, V_L_ 90–95, V_H_ 96–99 and V_H_ 101–110 [43,44,45]. Moreover, there is a hydrogen bond between an amino *H* provided from residue Arg 96 with atom *N*-1 of 7-ethylcamptothecin. However, hydrogen bonds exist generally between amino acid residues and analogues except for camptothecin, such as *N* of R_3_ side chain CH_2_N(CH_3_)_2_ with hydroxyl *H* of Ala-96, carbonyl *O* at C-21 with amino *H* of Asn-54, hydroxyl *H* at C-20 with hydroxyl *O* of Ala-53, hydroxyl *O* at C-20 with hydroxyl *H* of Tyr-33 for topotecan; carbonyl *O* at C-21 with amino *H* of Tyr 99, hydroxyl *O* at C-20 with amino *H* of Arg 96, β-*N* of R_2_ side chain with hydroxyl *H* of Glu 28 for irinotecan; *N*-1 with amino *H* of Arg 96 for 10-hydroxycamptothecin; *N*-1 with hydroxyl *H* of Tyr 98, carbonyl *O* at C-21 with amino *H* of Arg 96, hydroxyl *O* at C-20 with hydroxyl *H* of Tyr 94, carbonyl *O* at C-16a with hydroxyl *H* of Tyr 91 for SN-38. Furthermore, it may be the formation of hydrogen bond that induces the enthalpy playing important roles for the interaction of IgG with analogues, including topotecan, irinotecan, 10-hydroxycamptothecin and SN-38; as for 7-ethylcamptothecin, the formed hydrogen bond contributes a certain extent to the IgG interaction, though the main binding mode is hydrophobic interaction as camptothecin indicated by the results of binding mode study. However, it is remarkable that this hydrophobic antigen/semiantigen-binding cavity of IgG can enwrap drugs into host-guest inclusion complexes and prevent hydrolysis of the encapsulated drugs. Also, 7-ethylcamptothecin is adjacent to the IgG chromophores of Tyr 32, Tyr 33, Tyr 91, Tyr 94, Tyr 98 and Tyr 99, and may exchange energy with these chromophores of IgG such as Tyrs and quench the fluorescence of the protein [46,59,60], which is consistent with the results of fluorescence quenching study. Additionally, the docking scores calculated were −22.15 KJ/mol, −11.10 KJ/mol, −16.41 KJ/mol, −17.63 KJ/mol, −17.00 KJ/mol and −13.65 KJ/mol for camptothecin, topotecan, irinotecan, 10-hydroxycamptothecin, 7-ethylcamptothecin and SN-38, respectively, indicating the spontaneous interactions of IgG and CPTs, which is also consistent with the results of the thermodynamic study.

## 3. Materials and Methods

### 3.1. Materials

Standard samples of camptothecin analogues (purity ≥ 98%) was purchased from Sichua Ruibo Tech. Co., Ltd. (Nanchong, China), and human intravenous immunoglobulin (IVIG), content of protein 5%, IgG ≥ 95%, pH 4) was from Chengdu Rongsheng Pharmaceutical Co., Ltd. (Chengdu, China). Phosphate buffer solutions (PBS) of 0.1 M were selected to maintain the desired pH conditions. IVIG of 10.0 µM solution was prepared by diluting the original IVIG solution (*c*_IgG_ = 0.32 mM) by PBS at pH 4.0, in which the molecular weight of IgG was taken as 150 KD. 7-Ethylcamptothecin of 1.0 mM solution was prepared by dissolving the powder of standard sample in DMF, which were stored at 275~281 K until they were used.

### 3.2. Methods

Fluorescence spectrum were recorded on a RF-7000 spectrofluorophotometer (Hitachi, Honshu Island, Japan) with a constant temperature water bath. The binding constant (*K*_o_) were calculated by the Sips procedure as the following equations [59,60,61]:

(1) $$\frac{r}{n}=\frac{{\left(K_{o}c\right)}^{\alpha }}{1+{{(K}_{o}c)}^{\alpha }}\;$$

(2) $$\mathrm{lg}\frac{r}{n-r}=\alpha \mathrm{lg}c+\alpha \mathrm{lg}K_{o}\;$$

where *r* is moles of drug bound by per mole of protein, *c* is molar concentration of free drug, *n* is number of binding sites of per mole IVIG binding to drug, *K*_o_ is binding constant or average affinity constant, and α is affinity heterogeneity index of protein.

Fluorescent quenching mechanism was determined by Stern-Volmer equation [47,48]:

*F*_o_*/F* = 1 + *K*_q_τ_o_[*Q*] = 1 + *K*_SV_[*Q*] (3)

where *F*_o_ and *F* are fluorescence intensity in the absence and in the presence of drug at molar concentration of [*Q*], respectively, *K*_q_ is quenching rate constant of bimolecular diffusion collision, *K*_SV_ is Stern-Volmer dynamic quenching constant, τ_o_ is average fluorescence lifetime for biological macromolecule, and [*Q*] is molar concentration of drug.

Thermodynamic parameters were calculated upon temperature dependence of the average affinity constant for drug–IVIG binding. The temperatures used were 289, 296, 303 and 310 K. The enthalpy change (*H*^o^) was calculated from the slope and the entropy change (*S*^o^) was calculated from the intercept of Gibbs–Helmholtz equation:

ln*K*_o_ = −Δ*H*^o^/*RT* + Δ*S*^o^/*R* (4)

The free energy change (Δ*G*^o^) was calculated from the following relationship of Δ*H*^o^ (enthalpy) and Δ*S*^o^ (entropy):

Δ*G*^o^ = Δ*H*^o^ − *T*Δ*S*^o^ (5)

FT-IR spectra were recorded on a Nexus 670 FT-IR spectrometer (Nicolet, Madison, WI, USA) equipped with a germanium attenuated total reflection (ATR) accessory, a DTGS KBr detector and a KBr beam splitter, at room temperature. All spectra were taken via the ATR method with resolution of 4 cm^−1^ and 60 scans [62]. Each Lorentzian band was assigned to a secondary structure of protein based upon its maximum frequency: 1684–1696 cm^−1^ for β-antiparallel; 1662–1679 cm^−1^ for Turn; 1620–1638 cm^−1^ for β-sheet; 1654–1659 cm^−1^ for α−helix and 1640–1648 cm^−1^ for Random coil assigned as the remainders [63]. 

Molecular docking was carried out referring to the literature [43]. The drug structure was constructed with the Maestro [64] building tools. Then, building structure was preprocessed by the LigPrep [65] which used OPLS-2005 force field [66] and gave the corresponding low energy 3D conformers of the drug. The ionized state was assigned by Epik [67] at a target pH value of 7.0 ± 2.0. The 3D crystal structure of human IgG for molecular docking was retrieved from the Protein Data Bank (PDB ID code 1AJ7) [68]. The drug was docked into 5-(*p*-nitrophenylphosphonate)-pentanoic acid binding site of IgG by the Glide [69] with standard precision (SP) scoring mode. 

## 4. Conclusions

The binding performance of IVIG and 7-ethylcamptothecin is investigated in vitro by spectroscopy and molecular docking. Further the interaction properties of IVIG with other CPTs, including topotecan, irinotecan, camptothecin, SN-38 and 10-hydroxycamptothecin, are collected and compared each other. The binding of IVIG to these CPTs involves non-specific, spontaneous and weak interactions with binding constants of 10^3^~10^4^ M^−1^. Due to the different substituents on the *A*-ring or/and *B*-ring, entropy plays the main role for camptothecin and 7-ethylcamptothecin, while enthalpy plays the main role for other analogues. The hydrophobic antigen-binding cavity of IgG can enwrap drugs into host-guest inclusion complexes and prevent the hydrolysis of the encapsulated drugs. The typical β−sheet composition of IVIG decreases while the remainder, mainly random coil and α-helix, increases in the presence of CPTs. Moreover, IVIG may maintain higher levels of the lactone moieties of CPTs at physiological conditions in contrast to HSA. With its remarkable binding properties that may synergize with anti-cancer drugs, IVIG may be used as an important transporter protein for CPTs. In the future study and clinical application in vivo, an emulsion of drug:protein of 4:1 binding stoichiometry can be prepared, in which an 50 mL original IVIG solution (0.32 mM, 5% content of protein, IgG ≥ 95%, pH 4) contains 24.1 mg 7-ethylcamptothecin (Mr = 376 g/mol). Other emulsions of IVIG containing other CPTs can be prepared as the same method. However, more attention must be paid to the content (9.5~12.5 mg/mL or 0.063~0.083 mM) of IgG in adult serum, the commercial resource, antibody repertoire and dosage of IVIG, and guidelines of evidence-based IVIG and CPTs to improve patient safety and reduce the burden on healthcare systems [70,71].

## Figures and Tables

**Table 1:** Parameters of IVIG binding to six CPTs at pH 4.0 and different temperatures [43,44,45].

Temperature K	*K*_o_ × 10^4^/M^−1^	α	*K*_o_ × 10^4^/M^−1^	α	*K*_SV_ × 10^4^/M^−1^	*K*_q_ × 10^12^/M^−1^·s^−1^
	*n* = 2		*n* = 4		*n* = 2, *n* = 4	
CPT						
289	0.2636	0.4529	0.04152	0.4391	4.988	4.988
296	0.8933	0.5784	0.1968	0.5544	6.737	6.737
303	4.135	0.9097	1.645	0.8830	11.74	11.74
310	9.683	1.373	5.341	1.343	14.67	14.67
Topotecan chloride						
289	1.452	1.158	0.6501	1.099	4.743	4.743
296	1.528	1.101	0.6324	1.032	3.930	3.930
303	0.9268	0.8889	0.3119	0.8311	3.499	3.499
310	0.8570	0.7064	0.2028	0.6483	3.479	3.479
Irinotecan chloride						
289	1.500	1.061	1.790	0.2290	4.514	4.514
296	1.122	0.8402	1.371	0.1779	4.497	4.497
303	1.052	0.8245	1.306	0.1752	3.623	3.623
310	0.9247	0.8708	1.167	0.1873	3.376	3.376
10-Hydroxycamptothecin						
289	0.9805	0.9365	0.4065	1.118	3.155	3.155
296	0.8630	1.157	0.3477	0.8823	2.410	2.410
303	0.8587	0.9614	0.3347	0.9164	2.134	2.134
310	0.6337	0.8893	0.2304	0.8485	1.979	1.979
7-Ethylcamptothecin						
289	1.301	1.351	0.6763	1.300	2.744	2.744
296	0.6293	1.010	0.2702	0.9769	1.888	1.888
303	0.8375	1.087	0.3764	1.048	1.768	1.768
310	0.8472	1.161	0.4072	1.125	1.552	1.552
SN-38						
289	1.183	1.185	0.7992	1.284	1.923	1.923
296	1.088	1.308	0.5671	1.269	1.907	1.907
303	0.4756	0.8135	0.1633	0.7832	1.308	1.308
310	0.2731	0.8007	0.0973	0.7793	0.8997	0.8997

**Table 2:** Thermodynamic parameters of IVIG binding to six camptothecin analgues at pH 4.0 [43,44,45].

Temperature K	Δ*G*^o^/KJ·mol^−1^	Δ*H*^o^/KJ·mol^−1^	Δ*S*^o^/J·mol^−1^·K^−1^	Δ*G*^o^/KJ·mol^−1^	Δ*H*^o^/KJ·mol^−1^	Δ*S*^o^/J·mol^−1^·K^−1^
	*n* = 2			*n* = 4		
CPT, binding mode: hydrophobic interaction						
289	−13.39	131.5	501.4	−8.953	177.8	645.9
296	−16.72			−13.00		
303	−20.98			−18.66		
310	−23.66			−22.12		
Topotecan, binding mode: Van der Waals force, hydrogen bonding						
289	−23.03	−31.68	−27.49	−21.10	−44.49	−79.66
296	−23.71			−21.54		
303	−23.01			−20.27		
310	−23.34			−19.63		
Irinotecan, binding mode: electrostatic interaction						
289	−23.12	−16.21	23.43	−23.54	−14.26	31.70
296	−22.96			−23.45		
303	−23.34			−23.89		
310	−23.55			−24.15		
10-hydroxycamptothecin, binding mode: electrostatic interaction						
289	−22.08	−13.90	28.53	−19.97	−18.42	5.646
296	−22.30			−20.07		
303	−22.82			−20.44		
310	−22.56			−19.95		
7-ethylcamptothecin, binding mode: hydrophobic interaction						
289	−22.76	16.31	128.2	−21.19	22.45	141.9
296	−21.53			−19.45		
303	−22.76			−20.74		
310	−23.31			−21.42		
SN-38, binding mode: Van der Waals force, hydrogen bonding						
289	−22.53	−55.35	−112.1	−21.59	−80.35	−202.1
296	−22.87			−21.27		
303	−21.33			−18.64		
310	−20.39			−17.73		

**Table 3:** The secondary structural composition of free IVIG and its six camptothecin analogue complexes estimated by FT-IR difference spectra, self-deconvolution, second derivative resolution enhancement and the curve-fitting procedures of amide I band at 296 K. The concentration of IVIG is 2.0 µM [43,44,45].

Amide I components (%)	pH 4.0			pH 7.4		
	IVIG	+4.0 µM Analogues	+8.0 µM Analogues	IVIG	+4.0 µM Analogues	+8.0 µM Analogues
CPT						
β-anti	5.10	2.79	10.24	3.61	6.16	4.85
Turn	8.28	13.72	3.46	24.88	3.68	10.46
β-sheet	40.04	11.09	little	39.49	23.73	little
Remainders	46.58	72.40	~86.30	32.04	66.43	~84.69
Topotecan						
β-anti	5.10	little	8.93	3.61	4.45	little
Turn	8.28	~32.55	32.66	24.88	31.03	~18.28
β-sheet	40.04	15.72	3.22	39.49	24.26	27.73
Remainders	46.58	51.73	55.19	32.04	40.26	53.99
Irinotecan						
β-anti	5.10	little	little	3.61	6.16	little
Turn	8.28	~4.97	~6.78	24.88	29.19	~14.81
β-sheet	40.04	little	little	39.49	30.50	little
Remainders	46.58	~95.03	~93.22	32.04	40.31	~85.19
10-Hydroxycamptothecin						
β-anti	5.10	10.24	6.17	3.61	little	little
Turn	8.28	3.46	little	24.88	~16.00	~33.71
β-sheet	40.04	little	33.36	39.49	little	little
Remainders	46.58	~86.31	~60.47	32.04	~84.00	~66.29
7-Ethylcamptothecin						
β-anti	5.10	little	little	3.61	7.68	2.76
Turn	8.28	1.36	~16.93	24.88	21.28	16.64
β-sheet	40.04	29.98	26.30	39.49	15.82	7.57
Remainders	46.58	~68.66	56.77	32.04	55.22	73.03
SN-38						
β-anti	5.10	6.84	little	3.61	11.79	little
Turn	8.28	35.40	~30.23	24.88	5.19	~24.50
β-sheet	40.04	1.51	little	39.49	15.37	32.25
Remainders	46.58	56.25	~69.77	32.04	67.64	43.25

**Table 4:** The lactone-to-total ratios of 8.0 µM SN-38, 10-hydroxycamptothecin and topotecan after addtion of 2.0 µM IVIG within the subsequent incubated 60 min, at pH 7.40 and 310 K, respectively [43].

Compounds	Ratios of Lactone to Total for Three CPTs at pH 7.40 and 310 K	
SN-38	90.40%	86.47~87.35%
10-Hydroxycamptothecin	86.68%	56.56~68.58%
Topotecan	34.47%	29.76~32.71%
